# Predictive value of prognostic nutritional index for coronary heart disease: Evidence from a national population survey

**DOI:** 10.1515/jtim-2025-0058

**Published:** 2025-12-22

**Authors:** Yulu Jiang, Jiana Yang, Hanfei Wang, Qian Zhao, Jianquan Yu, Anru Cao, Lianying Guo, Jie Wu

**Affiliations:** School of Public Health, Shenyang Medical College, Shenyang, Liaoning Province, China; Liaoning Medical Functional Food Professional Technology Innovation Center, Shenyang Medical College, Shenyang, Liaoning Province, China; Mr. Selenium (Jilin) Biotechnology Co., Ltd., Shenyang, Liaoning Province, China; Shangshan Innovation Research (Shenyang) Technology Co., Ltd., Shenyang, Liaoning Province, China

## To the editor

One of the main causes of global burden of diseases is cardiovascular disease, specifically coronary heart disease (CHD). Traditional risk models, while useful, often lack integration of nutritional and immunological indicators. Recently, the predictive nutritional index (PNI) has developed as an innovative biomarker that reflects dietary status and immunological. This dual representation may offer new insight into chronic disease mechanisms, especially atherosclerosis and systemic inflammation.^[1]^ Recent work demonstrating the potential of biomarker-based approaches in CHD prognosis.^[2]^ This highlights the importance of exploring novel prognostic indicators such as PNI, which is easy to use, affordable, and accessible. Moreover, recent research reported trends in acute myocardial infarction-related mortality in the United States, revealing persistent racial and ethnic disparities and emphasizing the need for effective, equitable prognostic tools such as the PNI.^[3]^

With a sample size of 17,121 individuals aged 20 and above, the present analytical research employed data derived from the database of National Health and Nutrition Examination Survey (NHANES) 2008–2023. Inclusion criteria consisted of: age ≥ 20 years; availability of complete data for PNI computation, incorporating albumin levels and lymphocyte counts; and confirmed diagnostic information for CHD, ascertained *via* the question “Has a health professional told you that you have coronary heart disease?”. People who didn’t have all the information needed or had very unusual data were excluded from the research. PNI is calculated as serum albumin (g/L) plus 5 times the total number of lymphocyte (10^9^/L).

Covariate selection: Age, gender, race/ ethnicity, education, status in marriage, family income-to-poverty ratio (PIR), diabetes, hypertension, tobacco use, body mass index (BMI), total cholesterol (TC), and high-density lipoprotein cholesterol (HDL-C) were the covariates that were selected. First, the least absolute shrinkage and selection operator (LASSO) regression was used to find the candidate variables.

Statistical analysis: Two logistic regression models were created for statistical analysis. Model 1: A fully adjusted model that does not include PNI. Model 2: A model that has been fully corrected with PNI.

This research figured out 95% confidence intervals (CIs) and odds ratios (ORs). Prediction performance was assessed using area under the curve (AUC) and receiver operating characteristic (ROC) curves. All analyses were conducted in R version 4.3.3 utilizing the pROC package, with *P* < 0.05 considered statistically significant.

**Figure 1:**
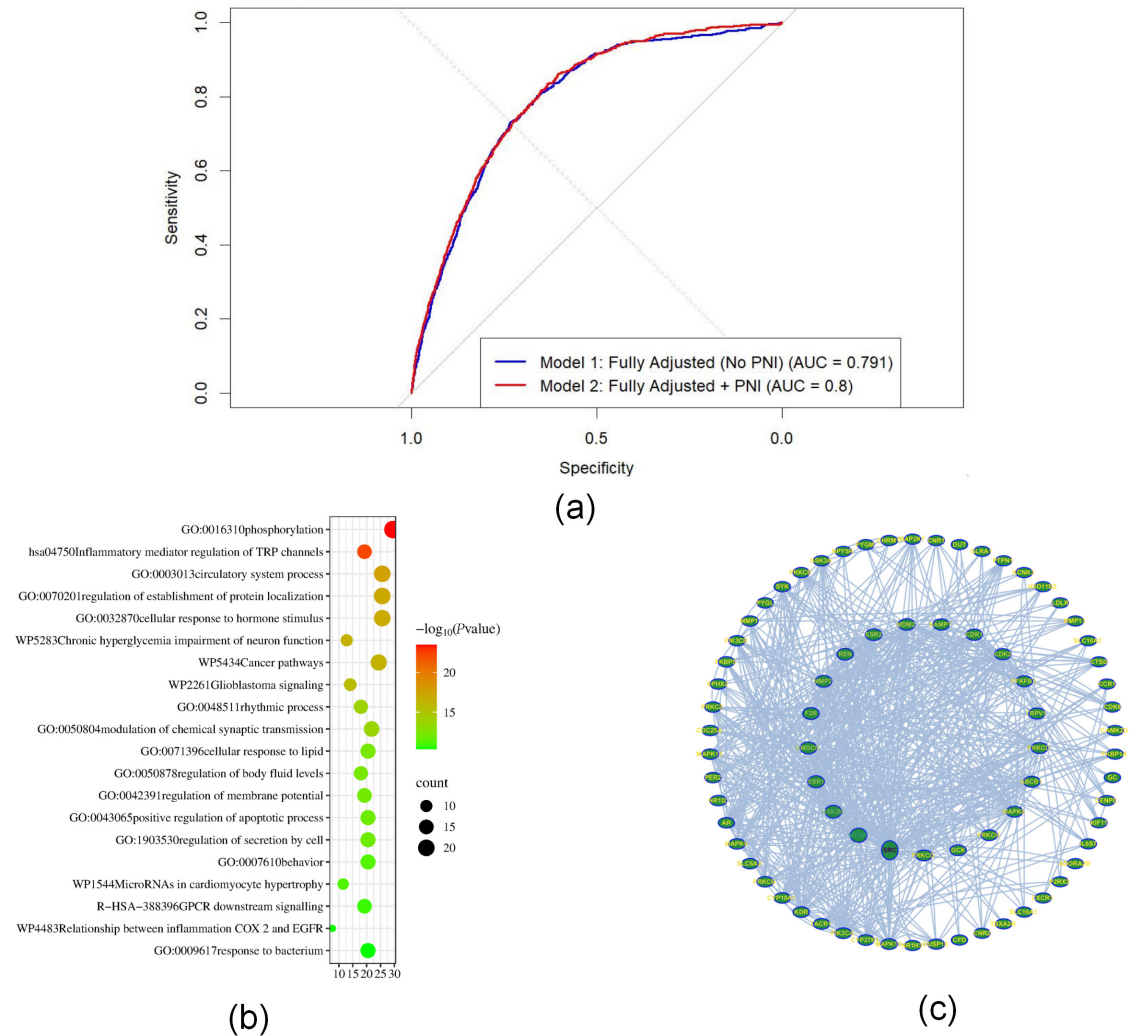
Composite visualization of predictive nutritional index (PNI)’s predictive value and biological relevance in coronary heart disease. (a) ROC curves that show the difference between fully adjusted logistic regression models with and without PNI. Adding PNI made the model work a little better (the AUC went from 0.791 to 0.800). (b) GO/KEGG enrichment analysis of PNI-associated genes indicated substantial participation in immune response and inflammation-related pathways. (c) Protein-protein interaction (PPI) network of key genes linked to PNI illustrating dense interactions within immune-regulatory modules.

Figure 1(a) shows the ROC curves that compare two models with progressive adjustment procedures to see if the PNI adds any prognostic value when it comes to CHD. The little rise in AUC from Model 1 (0.791) to Model 2 (0.800) implies that PNI may provide some more predictive information beyond standard risk factors. The AUC from the fully adjusted model with PNI indicates that PNI is still good at making predictions even when there are a lot of other things to think about.

Model 1 modified age, gender, race/ethnicity, educational attainment, marriage status, family income-to-poverty ratio, diabetes, hypertension, cigarette use, body mass index, total cholesterol, and high-density lipoprotein cholesterol. Model 2 adjusted the same variables just as Model 1 did, with the addition of PNI. In the fully adjusted model (Model 2), PNI continued to be a significant predictor of CHD. The odds ratio (OR) of PNI for CHD was 0.94 (95% CI: 0.92–0.95), with a *P*-value < 0.001 (Supplementary Table S1). This indicates that the probability of developing CHD decreased by around 5.7% with each unit increase in PNI, while accounting for additional variables such as gender, race or ethnicity, education, income, marital status, hypertension, diabetes, BMI, lipid profile, and other LASSO-selected covariates. These findings illustrate the autonomous predictive relevance of PNI beyond traditional cardiovascular risk factors.

We conducted GO and KEGG enrichment analyses and constructed a protein-protein interaction (PPI) network to investigate potential biological mechanisms linking PNI-related genes to coronary heart disease. The gene ontology results showed a lot of enrichment in biological processes like immune response, controlling inflammatory signals, and the movement of white blood cells. KEGG pathway analysis further demonstrated enrichment in atherosclerosis, cytokine-cytokine receptor interaction, and tumor necrosis factor signaling pathways. The protein–protein interaction (PPI) network analysis identified several hub genes involved in immune regulation and metabolic inflammation, perhaps clarifying the relationship between decreased PNI and increased CHD risk [Figure 1(b) and 1(c)].

Our results show that the PNI is still a good, noninvasive way to measure the risk of CHD, even when we remove laboratory biochemical variables from the model. In the multivariable logistic regression utilizing PNI quartiles, the lowest quartile exhibited a markedly diminished risks of CHD in comparison to the highest quartile (Q1 *vs*. Q4: OR = 0.943, 95% CI: 0.92–0.95, *P* < 0.001, Supplementary Table S1). The fully adjusted model without PNI had an AUC of 0.791, and after PNI was added, the AUC went up to 0.800 (Figure 1A). Even though the AUC improvement was small, this means that PNI provides predictive value beyond demographic, behavior, and comorbidity characteristics. The reliability of PNI’s statistical significance in multivariable models affirms its strength as an independent predictor following the correction for confounding variables.

As shown in the supplementary material, Supplementary Tables S2-4 and Supplementary Figures S1-4 provide additional analysis and visualizations that support the main findings. Supplementary Table S2 details baseline feature, Supplementary Table S3-4 show the subgroup interactions, Supplementary Figure S1-2 show the Protein-Protein Interaction Network Analysis and Functional Enrichment Analysis, while Supplementary Figures S3 and S4 display the interaction analysis and random forest modelbetween PNI and CHD risk, respectively. These materials further reinforce the study’s conclusions on PNI as a predictor for CHD.

These results corroborate previous research indicating PNI as an autonomous prognostic indicator.^[4]^ For example, a research has shown that PNI is linked to heart problems.^[5]^ Our results extend their findings by demonstrateing PNI’s performance across population and clinical adjustments. Functional enrichment and PPI studies indicated that PNI-related genes participate in immune response, inflammatory signaling, and atherosclerosis-associated pathways. These results substantiate a mechanistic association between diminished PNI and elevated CHD risk. It is important to note that the definition of PNI itself determines that it is significantly affected by the nutritional status of individuals. Because PNI is derived from albumin and lymphocyte counts, PNI values may be low in people with malnutrition, chronic inflammation, or other chronic diseases, even if there are no significant cardiovascular abnormalities. Therefore, the interpretation of PNI must incorporate complete clinical information.

Clinically, PNI offers a simple, cost-effective, and widely available index for CHD management. It could be included in regular CHD risk assessments, especially in places where resources are limited. Subsequent research ought to confirm the efficacy of PNI in longitudinal risk assessment across varied populations.^[6]^

Another implication of our findings pertains to prospective public health applications. Since albumin and lymphocyte counts are common blood tests, PNI can be figured out without any extra costs or tests.^[7]^ In areas with limited resources when complete cardiovascular evaluations are impractical, PNI may function as an effective surrogate indication for stratifying CHD risk.

In clinical practice, PNI is a straightforward and economical index derivable from standard blood tests; yet, our findings indicate that it maintains predictive significance even in the absence of explicit adjustments for laboratory biochemical markers. Combining PNI with demographic and clinical characteristics as well as habits could provide a more balanced way to measure risk. Future research should focus on testing these kinds of models in other groups of people.^[8,9]^

In conclusion, the prognostic nutritional index shows a steady, moderate capacity to predict CHD. Lower PNI levels were strongly correlated with an increased risk of CHD, and the inclusion of PNI somewhat enhanced the prediction efficacy of a fully adjusted non-laboratory model. Given its simplicity and accessibility, PNI could be a practical tool for CHD risk stratification, especially in resource-limited settings.

## Supplementary Information

Supplementary materials are only available at the official site of the journal (www.intern-med.com).

## Supplementary Material

Supplementary Material Details
