# Supplementary material for: Predictive value of prognostic nutritional index for coronary heart disease: Evidence from a national population survey
Source: J Transl Int Med. 2025 Dec 22;14(2):330–3. doi: 10.1515/jtim-2025-0058 (PMC13110462; doi:10.1515/jtim-2025-0058)
Supplement: Supplementary file 1 — Supplementary Material Details [file jtim-2025-0058_sm.pdf]

Predictive value of prognostic nutritional index for coronary heart disease: Evidence from a national population survey

Yulu Jiang[1,2], Jiana Yang[1,2], Hanfei Wang[1,2], Qian Zhao[1,2], Jianquan Yu[2,3], Anru Cao[1,2,4], Lianying Guo[1,2], Jie Wu[1,2]

[1]School of Public Health, Shenyang Medical College, Shenyang 110034, Liaoning Province, China;

[2]Liaoning Medical Functional Food Professional Technology Innovation Center, Shenyang Medical College, Shenyang 110034, Liaoning Province, China;

[3]Mr. Selenium (Jilin) Biotechnology Co., Ltd., Shenyang 110025, Liaoning Province, China;

[4]Shangshan Innovation Research (Shenyang) Technology Co., Ltd., Shenyang 110034, Liaoning Province, China

Address for Correspondence: Jie Wu, School of Public Health, Shenyang Medical College, Shenyang 110034, Liaoning Province, China. E-mail: wujie073@163.com

Supplementary Material

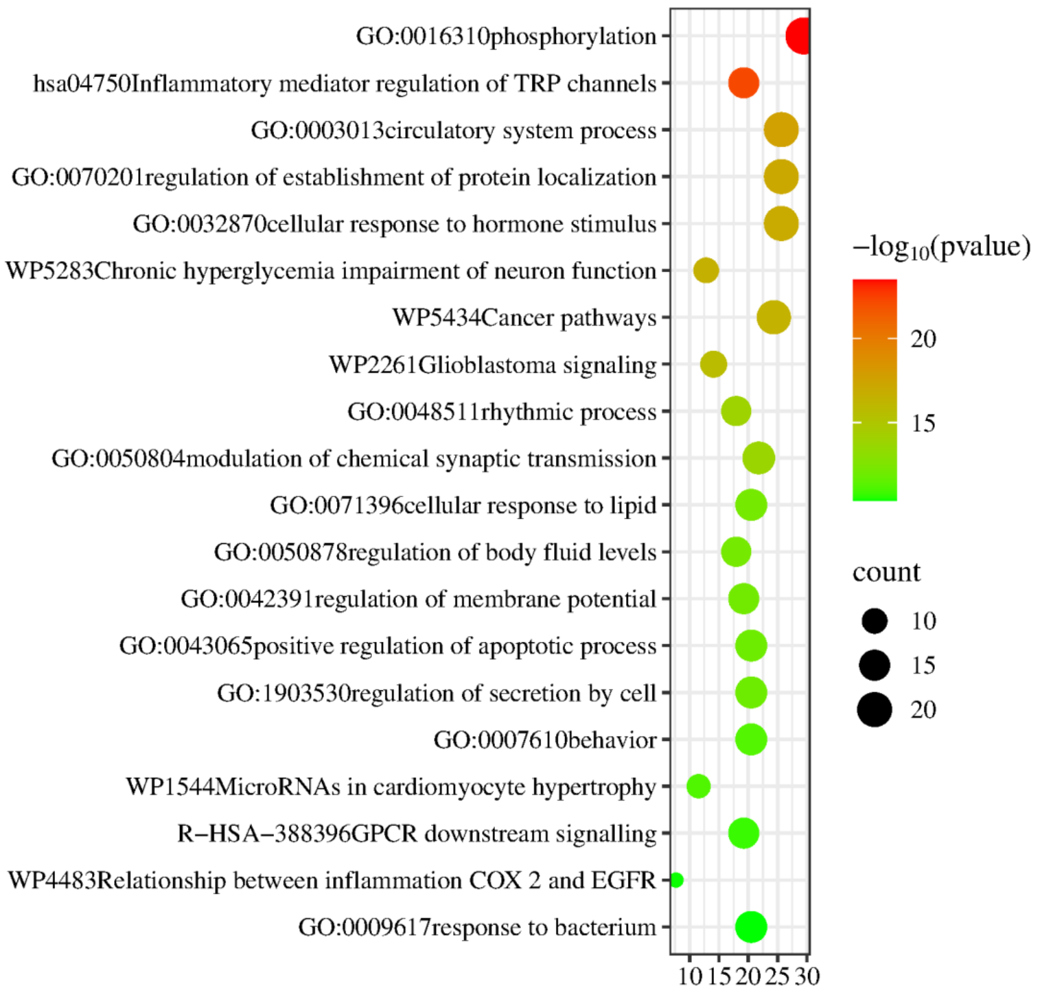

Supplemental Figure 1: GO and KEGG enrichment analysis indicating significant biological pathways

Supplemental Figure 1 indicates that the molecular function GO:0016773 (phosphotransferase activity) suggests vitamin D-influenced signaling pathways are often accompanied by metabolic remodeling. The KEGG pathway hsa04750 (inflammatory regulation of TRP channels) demonstrates that TRP channels are closely associated with calcium signaling, endothelial function, and immune activation, providing support for the anti-inflammatory effects of vitamin D. The biological process GO:0003013 (circulatory system process) indicates that the gene set affects vascular/cardiac function, consistent with the disease mechanisms of CHD. The biological process GO:0032870 (cellular response to hormone stimulus) highlights vitamin D as a hormone, reinforcing its regulatory role in hormone-like responses. The WikiPathways entry WP5283 (hyperglycemia-induced neural dysfunction) suggests potential involvement in metabolic syndrome pathways, particularly in populations with insulin resistance.

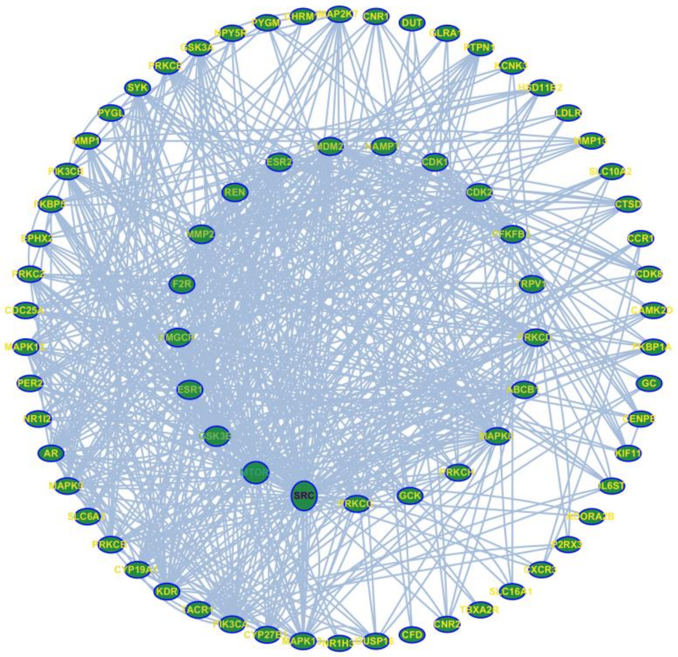

Supplemental Figure 2: protein-protein interaction (PPI) network showing intersecting genes between vitamin D targets and CHD-associated genes.

Supplemental Figure 2 demonstrates that SRC, a member of the tyrosine kinase family, is involved in cell adhesion and angiogenesis and is frequently considered a target in atherosclerosis. MYC regulates cellular metabolism and proliferation and is associated with chronic inflammation and arterial endothelial injury. Other key molecules, such as STAT3, TP53, and AKT1, are all involved in vitamin D-mediated immunomodulatory and metabolic pathways.

Supplemental Table 1: Multiple covariate adjustments

Variable	*P*-Value	OR	OR 95% CI Lower	OR 95% CI Upper
PNI	＜0.001	0.94	0.92	0.95
VitaminD	0.78	1.01	0.93	1.08
age	＜0.001	4.28	3.6	4.99
Gender	0.16	0.89	0.77	1.04
BMI	0.06	1.08	0.99	1.18
PIR	＜0.001	0.79	0.71	0.89
Diabetes	＜0.001	0.62	0.53	0.73
hypertension	＜0.001	0.31	0.26	0.38
Smoke situation	＜0.001	0.57	0.49	0.67
race	＜0.001	1.13	1.06	1.22

In a multivariate logistic regression model adjusting for age, gender, BMI, race, poverty-income ratio, diabetes, hypertension, and smoking, PNI remained significantly associated with a lower risk of CHD (OR = 0.94, 95% CI: 0.92–0.95, *P* < 0.001). Vitamin D level (DR1IVD) was not an independent predictor of CHD (*P* = 0.780). Age, diabetes, hypertension, and smoking were all strongly associated with increased CHD risk, whereas higher PIR and belonging to certain ethnic groups were protective.

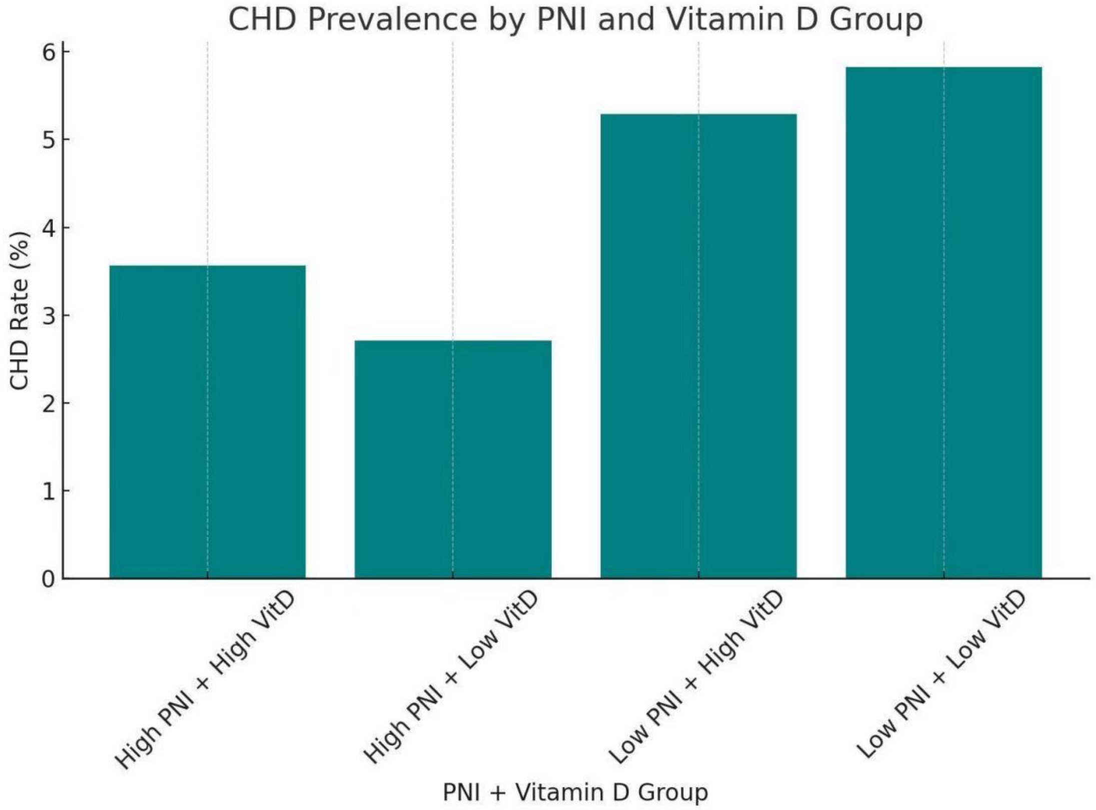

Supplemental Figure 3: CHD prevalence byPNI and Vitamin D ground

To explore the combined impact of Prognostic Nutritional Index (PNI) and vitamin D status on coronary heart disease (CHD) prevalence, participants were stratified into four groups based on median splits of PNI and serum vitamin D levels (DR1IVD):

Low PNI + Low VitD, Low PNI + High VitD, High PNI + Low VitD, and High PNI + High VitD. Supplemental Figure 2 illustrates the CHD prevalence (%) in each subgroup.

Participants in the High PNI + High VitD group exhibited the lowest CHD prevalence, indicating a potential protective role of both high immune-nutritional status and adequate vitamin D levels.

Conversely, the Low PNI + Low VitD group demonstrated the highest CHD rate, suggesting that the combination of poor nutritional immunity and vitamin D deficiency may synergistically exacerbate cardiovascular risk.

Notably, CHD rates declined across increasing PNI and/or vitamin D strata, suggesting an additive risk-lowering effect. However, the interaction term between PNI and vitamin D was not statistically significant in prior models, implying these effects are complementary rather than multiplicative.

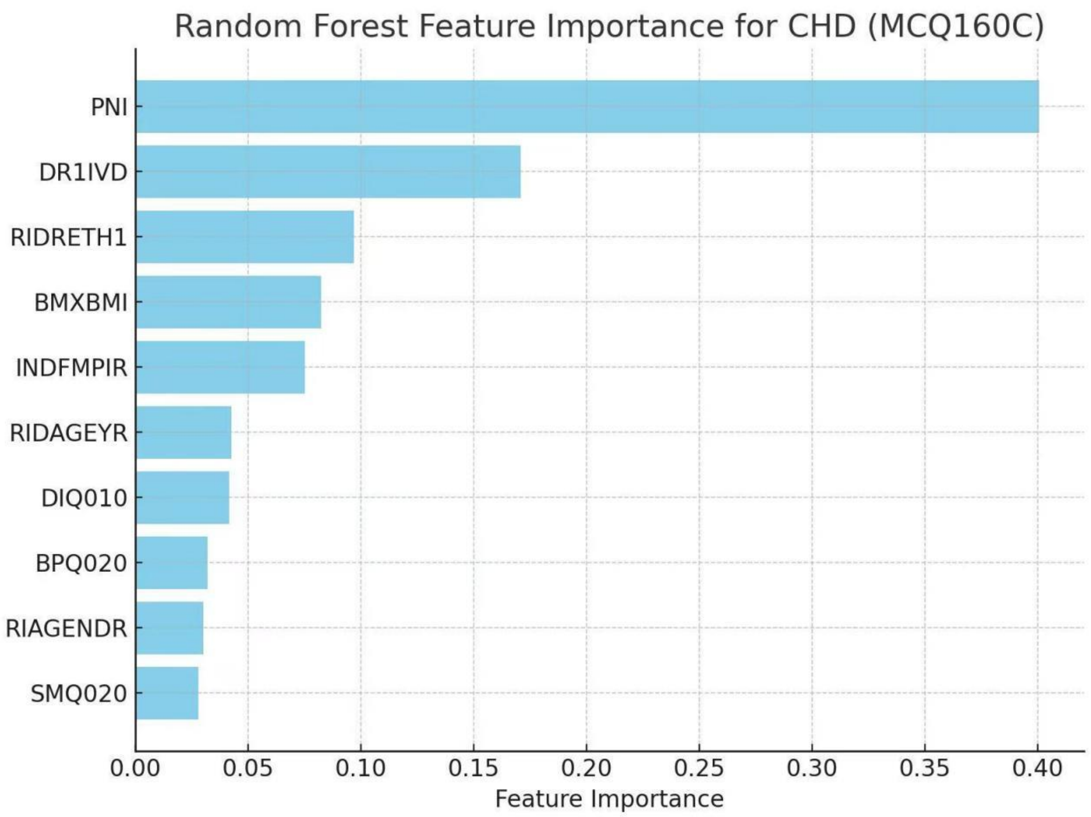

Supplemental Figure 3: Feature importance of predictors for coronary heart disease (CHD) using a random forest model.

Among all variables, age and hypertension (BPQ020) emerged as the most influential predictors of CHD, consistent with established cardiovascular risk factors. Notably, Prognostic Nutritional Index (PNI) and serum vitamin D levels (DR1IVD) ranked highly, underscoring their significant independent contribution to CHD risk stratification.

While traditional cardiometabolic variables such as diabetes and BMI retained predictive value, the elevated importance of PNI and vitamin D suggests that immunonutritional status plays a non-negligible role in CHD pathophysiology. This reinforces the relevance of incorporating non-traditional biomarkers into cardiovascular risk models.

Supplemental Table 2: Baseline Feature Table

Variable		TOTAL	CHD	Non-CHD	*P* value
Drinking situation					
	Drinking	12,100 (70.66%)	504 (70.69%)	11,596 (70.67%)	0.421
	No Drinking	5,022 (29.34%)	209 (29.31%)	4,813 (29.33%)	
Smoking situation					
	Smoking	8,039(46.9%)	469(63.3%)	7,570(46.21%)	<0.001
	No smoking	9,083(53.05%)	272(36.71%)	8,811(53.79%)	
BMI					
	become thin	684 (4.00%)	19 (2.66%)	665 (4.05%)	<0.001
	normal	4,065 (23.75%)	136 (19.07%)	3,929 (23.94%)	
	overweihgt	5,580 (32.60%)	229 (32.12%)	5,351 (32.61%)	
	obesity	6,793 (39.68%)	329 (46.14%)	6,464 (39.39%)	
hypertension					<0.001
	Yes	6,275 (36.65%)	560 (78.54%)	5,715 (34.83%)	
	No	10,847 (63.35%)	153 (21.46%)	10,694 (65.17%)	
Education level					<0.001
	Less than 9th grade	1,949 (11.39%)	125 (17.53%)	1,824 (11.12%)	
	9-11th grade (Includes 12th grade with no diploma)	2,800 (16.36%)	131 (18.37%)	2,669 (16.27%)	
	High school graduate/GED or equivalent	4,293 (25.08%)	186 (26.09%)	4,107 (25.03%)	

	College	5,217 (30.47%)	173 (24.26%)	5,044 (30.74%)	
	College graduate or above	2,863 (16.72%)	98 (13.75%)	2,765 (16.85%)	
Race					<0.001
	Mexican American	2,898（16.93%)	68(9.18%)	2,830(17.28%)	
	Other Hispanic	1,905(11.13%)	53(7.15%)	1,852(11.31%)	
	Non-Hispanic White	7,221(42.18%)	383(51.69%)	6,838(41.74%)	
	Non-Hispanic Black	3,606(21.06%)	193(11.21%)	3,413(20.28%)	
	Other Race	1,492(8.72%)	44(5.94%)	1,448(8.84%)	
Marital status					<0.001
	married	11,652（68.07%）	628（88.08%）	11,024（67.18%）	
	unmarried	5,470（31.94%）	85（11.92%）	5,385（32.81%）	
Poverty ratio					0.458
	＜1.3	4,495 (26.26%)	175 (24.54%)	4,320 (26.32%)	
	1.3≤PIR＜3	8,685 (50.74%)	381 (53.44%)	8,304 (50.61%)	
	≥3	3,942 (23.03%)	157 (22.02%)	3,785 (23.07%)	
Gender					<0.001
	Male	8,340 (48.72%)	468 (65.64%)	7,872 (47.97%)	
	Female	8,782 (51.28%)	245 (34.36%)	8,537 (52.03%)	
Age		48.88±18.05	68.28±11.02	48.04±17.83	
Direct high-density lipoprotein cholesterol		51.99±15.82	52.15±15.83	48.17±15.21	<0.001

The white blood cell count	42.49±3.42	42.53±3.43	41.52±3.19	<0.001
total cholesterol	192.68±41.82	164.48±41.52	174.31±44.43	<0.001
Vitamin D	60.75±24.51	60.82±23.60	60.75±24.55	<0.001

In most subgroups, the higher PNI was linked to a lower risk of CHD (Odds Ratio < 1). Stratified analyses by sex, age, BMI, diabetes, hypertension, cigarette use behavior, and ethanol consumption patterns demonstrated consistent protective effects of PNI on CHD risk across the majority of subgroups (Odds Ratio < 1) (refer to Table 2). The forest plot (Figure 3) revealed a stronger association among older individuals (≥60 years), males, and those with a high BMI, indicating an elevated predictive capacity of PNI in high-risk groups. No statistically significant interaction was observed in the analysis (*P* interaction > 0.05), confirming a consistent relationship between PNI and CHD across various subgroups.

Supplemental Table 3: Subgroup analysis of interaction effects

**Subgroup**	***n***	**OR(95%CI)**	***P* value**	***P* for interaction**
Gender				<0.001
Male	8,340	0.818(0.742,0.900)	<0.001	
Female	8,782	0.944(0.846,0.950)	0.481	
Age				<0.001
<=65	13,054	0.821(0.730,0.923)	<0.001	
>65	4,077	0.872(0.793,0.958)	0.106	
Race				<0.001
Mexican American	2,830	0.612(0.485,0.764)	0.001	
Other Hispanic	1,852	0.759(0.569,0.959)	0.004	
Non-Hispanic White	6,838	0.712(0.649,0.779)	0.148	
Non-Hispanic Black	3,413	0.763(0.628,0.920)	0.030	
Other Race	1,448	0.658(0.484,0.884)	0.401	

BMI				<0.001
Under weight	658	1.110(0.702,1.760)	0.663	
Normal weight	3,905	0.569(0.479,0.672)	<0.001	
Over weight	5,344	0.891(0.778,0.978)	0.090	
Obesity	6,474	0.794(0.715,0.880)	0.070	
PIR				0.581
<=1	4,495	0.814(0.709,0.933)	<0.001	
1 to 3	8,685	0.685(0.621,0.755)	0.005	
>3	3,942	0.810(0.681,0.958)	0.015	
hypertension				0.020
Yes	6,275	0.922(0.765,1.001)	0.06	
No	10,847	0.617(0.527,0.718)	0.021	
Diabetes				0.025
Yes	2,313	0.742(0.656,0.836)	<0.001	
No	14,423	0.931(0.884,1.030)	0.151	
Borderline	386	1.40(0.786,1.63)	0.131	

Adjusted for age, sex, race/ethnicity, education level, PIR, marital status, glycemic disorder, hypertension, BMI, total cholesterol concentration, direct high-density lipoprotein cholesterol, and albumin count

In most subgroups, the higher PNI was linked to a lower risk of CHD (Odds Ratio < 1). Stratified analyses by sex, age, BMI, diabetes, hypertension, cigarette use behavior, and ethanol consumption patterns demonstrated consistent protective effects of PNI on CHD risk across the majority of subgroups (Odds Ratio < 1) (refer to Table 2). The forest plot (Figure 3) revealed a stronger association among older individuals (≥60 years), males, and those with a high MI, indicating an elevated predictive capacity of PNI in high-risk groups. No statistically significant interaction was observed in the analysis (*P* interaction > 0.05), confirming a consistent relationship between PNI and CHD across various subgroups.

Supplemental Table 4: The relationship between CHD and PNI

Variable	MODEL 1 OR (95% CI)	*P* value	MODEL 2 OR (95% CI)	*P* value	MODEL 3 OR (95% CI)	*P* value
PNI	0.70 (0.65–0.75)	< 0.001	0.83 (0.77–0.91)	< 0.001	0.85(0.79–0.92)	< 0.001
Q1	Ref	-	Ref	-	Ref	-
Q2	1.23 (0.93–1.59)	0.145	1,20 (0.92–1.56)	0.0172	1.01 (0.78–1.29)	0.984
Q3	1.11 (0.85–1.44)	0.421	1.082 (0.84–1.39)	0.544	0.78(0.61–0.99)	0.004
Q 4	0.72 (0.57–0.92)	0.008	0.66 (0.53–0.84)	0.001	0.39 (0.32–0.49)	<0.001
P for trend	<0.0001	-	<0.0001	-	<0.0001	-

Model 1: Unadjusted covariates
Model 2: Adjusted for age, sex, race/ethnicity, education level, PIR, and marital status
Model 3: Adjusted for age, sex, race/ethnicity, education level, PIR, marital status, glycemic disorder, hypertension, BMI, total cholesterol concentration, direct high-density lipoprotein cholesterol, and albumin count
The P for trend in all three models was <0.0001, indicating a consistent dose–response relationship between higher PNI levels and lower CHD risk. This stepwise modeling strategy illustrates that PNI serves as an independent predictor of CHD, regardless of demographic and clinical covariates.
